# Supplementary material for: Are carbon emissions trading and green financial instruments synergistic? -Comprehensive quantitative research based on content analysis
Source: PLoS One. 2024 Mar 7;19(3):e0298601. doi: 10.1371/journal.pone.0298601 (PMC10919601; doi:10.1371/journal.pone.0298601)
Supplement: S1 Data — (ZIP) [file pone.0298601.s001.zip › Data/地级市Z_F工作报告.zip!/║■▒▒/╗╞╩»╩╨/└┤╫╘╣1⁄2╓┌║┼í╛┬φ┐╦╩2╛▌═°í┐_2013.docx]

各位代表：

　　现在，我代表市人民政府向大会作政府工作报告，请予审议，并请市政协委员和列席人员提出意见。

　　一、2012年，全市上下齐心协力、砥砺奋进，经济社会实现平稳较快发展

　　过去的一年，我们坚持以科学发展观为指导，深入实施“三大战略”，成功举办湖北黄石第三届乒乓节暨第二届矿冶文化旅游节，国民经济和社会发展取得新成就。

　　——经济平稳快速增长。主要经济指标增幅高于、好于全省平均水平。初步预计，地区生产总值突破千亿元大关、达到1040亿元，比上年增长12%；地方财政总收入突破百亿元大关、达到113.18亿元，增长22.8%；公共财政预算收入完成65.06亿元，增长20.8%；金融机构存款余额突破千亿元大关。大冶市进入全国百强县市行列。

　　——“双迎”活动取得新成效。坚持以节会促开放促发展，全方位宣传推介黄石，进一步提升城市形象，巩固黄石矿冶名城、国乒基地的地位，让黄石走向世界，让世界了解黄石。

　　——改革创新取得重大突破。各项国家级试点工作稳步推进。成功创建全国创业先进城市。黄石保障性住房建设经验在全国交流。黄石工矿废弃地综合开发试验区获批。有色公司在香港成功上市，黄石上市企业数量位居全省第三位。大部制扁平化改革深入推进，社区网格化管理格局基本形成。

　　——社会事业加快发展。科技创新取得新成果，教育事业继续发展，公共卫生体系建设得到加强，文化、体育事业进一步繁荣。

　　——人民生活有较大改善。城镇居民人均可支配收入预计达到19200元，居全省第二位，农民人均纯收入达到7400元，分别增长13%和14%。市政府承诺的十件实事除第八件实事中的两个子项目未达到目标进度外，其他各项实事全部完成。

　　这些成就，标志着黄石综合实力进一步增强，我们朝着全面建成小康社会目标迈出坚实的一步。

　　一年来的主要工作是：

　　（一）坚持以双迎活动为契机，着力提升城市知名度、美誉度和开放度

　　全力推进招商引资和项目建设。以节会为平台，统筹抓好产业招商、小分队招商、专题招商，全市招商引资新签合同项目106个，总投资473亿元，增长34.7%。扎实开展重大项目建设年活动，推进6大类、113个重点项目建设，全市完成固定资产投资750亿元，增长25.5%。航天电缆产业园、合兴纺织一期等74个亿元项目建成投产，沪士电子、奥莱斯全钢子午轮胎等170个重大项目开工建设，新兴管业球墨铸管升级改造、武汉重冶模坯制造等78个续建项目加快推进。积极争取中央和省支持矿山地质环境治理、整装勘查等项目资金12.29亿元。

　　着力改善城市面貌。实施道路改造、场馆建设、宾馆改造升级、环境综合整治等工程，推进沿湖路等10条主次干道综合改造，新增及改造城区道路里程38.4公里；新黄石一中体育馆建成投入使用，中国乒乓球队黄石训练基地完成主体工程，磁湖山庄等16家星级宾馆改造升级；建成磁湖东南岸等一批景观工程，新增绿地面积64.9公顷；完成14条主要迎宾线路立面整治和主干道五化成片整治，对40个社区、13个农贸市场、150条背街小巷进行了整体改造，美化亮化楼宇1000余栋，城市环境面貌焕然一新。

　　进一步扩大黄石影响力。成功举办世界杯女子乒乓球赛、中国发展论坛等8大类15项主题活动，赢得海内外的广泛赞誉，开启黄石节会国际化的新阶段。着力打造黄石名片，先后荣获中国观赏石之城、中国男装名城、中国挤出模具之都、湖北省历史文化名城称号。

　　（二）坚持以转变发展方式为主线，推进资源型城市转型跨越

　　深入推进产业转型升级。实施亿元以上产业转型项目150个，黑色金属、有色金属等传统产业竞争力明显提升，装备制造、纺织服装等优势潜力产业不断壮大，电子信息、节能环保等战略性新兴产业加快发展，全市省级重点成长型产业集群达到4个。实施中小企业成长工程，新增规模工业企业86家。着力发展现代服务业，新兴际华物流等一批重点项目顺利实施，黄石港区获批全省服务业综合改革示范区，第三产业完成增加值310亿元，增长8.7%。

　　加快推进生态文明建设。实施开山塘口整治、矿山复垦、石漠化治理等生态修复工程，完成黄荆山北麓、板岩山治理等项目，新增造林面积5.37万亩，生态环境逐步改善。建成大冶有色铜冶炼余热发电、尖峰水泥脱硝等重点节能减排项目，华新水泥、大江集团被国家列为首批资源综合利用“双百工程”[1]骨干企业，万元生产总值能耗及主要污染物减排完成省定目标，全年国家二级标准以上天数达到346天，是黄石有监测记录以来的最好水平。集中式饮用水源水质达标率100%，磁湖水环境综合治理工程启动实施，大冶湖保护工作进一步加强。

　　统筹推进社会事业协调发展。市级财政投入各项社会事业11.8亿元，增长43.2%。统筹发展各级各类教育，义务教育均衡发展示范县（市、区）创建工作稳步推进，新黄石一中建成使用，黄石理工学院更名湖北理工学院，湖北师范学院更名湖北师范大学筹备工作取得进展，高校债务化解成效明显。医药卫生体制改革不断深化，启动大冶市人民医院公立医院改革，县、乡、村三级卫生服务体系逐步完善。积极开展国家公共文化服务体系示范区创建工作，加快公共文化设施建设，建成镇、村（社区）文化站（室）600家，农家书屋864家。加强文化遗产保护，黄石矿冶工业遗产列入中国世界文化遗产预备名单。着力构建和完善全民健身服务体系，群众体育和竞技体育水平同步提升。广播电视事业蓬勃发展。继续稳定低生育水平，人口和计划生育工作取得新成绩。

　　积极推进改革创新。两型社会建设综合配套改革不断深化，黄石进入《全国老工业基地调整改造规划》和全国工矿废弃地复垦利用、全国构建国土资源管理新格局试点城市行列。住房保障国家级“双试点”[2]改革深入推进。融资体制改革实现新突破，市城投公司12亿元二期企业债券顺利发行，磁湖水环境综合治理利用亚行贷款项目获批。集体林权制度改革主体任务基本完成。深化行政审批制度改革，取消行政审批54项，减少审批环节1200个。科技创新能力进一步增强，新增国家级企业技术中心1家，高新技术企业20家，新增省级工程技术研发中心3家，创新型企业4家，专利授权量首次突破1000件。

　　（三）坚持以统筹城乡发展为方向，推进新型城镇化和城乡一体化

　　整体提升区域经济发展水平。制订落实支持县（市）区加快发展相关政策，统筹抓好各级各类工业园区开发建设。黄金山工业新区实施东扩南延，20平方公里起步区基础设施日益完善，引进光谷联合科技城等7个10亿元以上项目；新港物流（工业）园基础设施建设全面启动。大冶经济开发区、阳新经济开发区、灵成工业园扩园步伐加快。全市园区基础设施投入完成95亿元，新引进项目资金430亿元。

　　完善城乡交通设施。全年完成交通基础设施投资52亿元，大广高速黄石段建成通车，全市高速公路从两年前的零里程增加到150公里，每百平方公里拥有高速公路里程达到东部发达地区平均水平。黄石物流中心、棋盘洲港区二期工程开工建设，武黄城际铁路黄石段、黄咸高速大冶段等重点工程加快实施。建成一、二级公路及农村公路407公里。积极争取上级支持，在十二五规划基础上，新增交通投资计划67亿元，新规划国、省道8条386公里。大冶市被列为全省通组公路试点县（市）。

　　巩固农业基础地位。全面落实党的强农惠农富农政策，深化农业结构调整，实施高产农田建设12.93万亩，粮食总产量实现九连增，农林牧渔业总产值120亿元，增长4.5%。现代农业加快发展，新增市级龙头企业8家，种植规模50亩以上的大户突破2000户，中粮、雨润、永大等农业产业化龙头企业发展规模不断壮大，万头养猪场达到23个，新增农民专业合作社238家，农村经济发展步入新的阶段。

　　推进城镇化和新农村建设。全面完成城镇总体规划修编工作，大力推进城镇基础设施建设，全市城镇化率达到59.5%。镇域经济实力不断增强，保安镇获批全国小城镇发展改革试点，7个乡镇进入全省百强乡镇行列。扎实开展万名干部进万村挖万塘活动，全市共完成塘堰整治及小型水利设施建设1.1万处；实施农村安全用水工程，解决了13.4万农村人口饮水不安全问题。着力抓好灵乡、浮屠、大箕铺、大王四个镇新农村建设试点，农村生产条件和村庄环境进一步改善，农村面貌发生新的变化。

　　（四）坚持以保障和改善民生为根本目的，切实维护广大人民群众的切身利益

　　扎实抓好创业带就业工作。落实各项帮扶政策措施，发放小额担保贷款4.81亿元，帮扶成功创业6850人，带动就业2.79万人；帮助城镇困难人员再就业1.6万人，转移农村富余劳动力2.32万人，组织就业技能培训2.44万人，有效缓解了就业难题。

　　完善社会保障体系。社会保障覆盖范围继续扩大，全市五项社会保险参保人数达到256.49万人（次）。城乡居民养老保险实现全覆盖，全市18万城乡居民基础养老金按时足额发放，适龄人员参保率达到91.7%。全民医保进程加快，全市城镇医保和农村新农合参保率分别达到96.5%和99.1%，在全省率先实行医疗保险城乡一体化改革，城区3万余名参合农民实现向城镇居民医疗保险制度转轨，农村居民医保待遇水平大幅提高。加强城乡低保工作，城区居民最低生活保障标准提高到420元/月，农村居民最低生活保障标准提高到2000元/年。保障性安居工程建设取得新突破，全市开工各类保障房19598套，启动城市棚户区改造项目33个、10634户，竣工各类保障房10534套，新增租赁补贴2421户。

　　依法维护社会稳定。加强和创新社会管理，推进平安黄石、法治黄石建设，严厉打击各种刑事犯罪活动，加强矛盾纠纷排查调处工作，群众安全感进一步提升。强化安全生产和食品药品监管，完善公共突发事件应急管理机制，成功处置突发性霍乱疫情，受到国家卫生部的充分肯定和省卫生厅的通报嘉奖。加强医疗纠纷调解工作，积极构建和谐医疗环境。抓好物价监测预警，落实社会救助和保障标准与物价上涨挂钩联动机制，发放价格临时补贴782.3万元，惠及困难群众50.5万人（次）。

　　与此同时，切实加强政府自身建设，坚持依法行政，自觉接受市人大及其常委会的法律监督、工作监督和市政协的民主监督，认真办理人大代表建议案和政协提案。党风廉政建设责任制全面落实，政风行风评议、行政监察和审计监督工作不断加强。积极开展文明创建活动，公民思想道德素质进一步提高。加强双拥工作，支持军队建设，军政军民关系更加密切。对台、保密、档案、妇女儿童、残疾人、老龄、援藏、援疆、援利川、人力资源、统计、工商、质监、外事、侨务、民族、宗教、国家安全、编制、科普、防震减灾、民防、水文、气象、史志等各项工作都取得新的成绩。

　　各位代表！回首过去的一年，我们在挑战中抢抓机遇，在困难中奋勇前行，取得了可喜的成绩，这是市委正确领导、全市人民顽强拼搏的结果，也是全市多年来接力奋斗、厚积薄发的结果。在此，我代表市人民政府，向全市广大干部群众，向给予政府工作大力支持和有效监督的人大代表、政协委员和各民主党派、工商联、无党派人士，向中央、省各驻黄单位和广大外来投资者、建设者，向驻黄解放军、武警官兵，向所有关心支持黄石建设和发展的同志们、朋友们，表示衷心地感谢！

　　我们也清醒地看到，我市经济社会发展仍存在不少困难和问题，主要是：经济总量小，结构不优，服务业发展滞后，新兴产业发展不快；市场主体发展不够，引进大项目、高新技术项目、外向型项目不多；节能减排、生态环境治理任务艰巨，转变经济发展方式的制约因素仍然较多；城乡低收入群体持续增收难度增大，部分地区发展基础薄弱；发展环境不优，办事效率不高，政府执行力有待进一步提升，等等。我们将直面人民群众的关切，正视这些问题，采取有效措施，认真加以解决。

　　二、积极实施三年行动计划，为提前全面建成小康社会打下坚实基础

　　未来三年，是黄石落实十二五规划、实现转型跨越的关键时期，是黄石提前全面建成小康社会[3]的决定性三年。纵观国际国内形势，黄石经济社会发展既面临难得的机遇，也面临严峻的挑战，但总体上机遇大于挑战，仍处于大有可为的重要战略机遇期。我们要抢抓机遇、应对挑战，自我加压、奋力赶超，到2015年建市65周年之际，基本建成小康社会，再通过1－2年的努力，确保与全省同步全面建成小康社会。

　　按照提前全面建成小康社会的总体要求，未来三年我们的奋斗目标是：

　　－－综合经济实力显著增强。到2015年，全市国内生产总值、人均生产总值在2010年的基础上翻一番；全社会研发投入占地方生产总值的比重大幅提高，进入国家创新型城市行列；大冶市在全国百强、阳新县在全省县域排位中稳步进位、各城区在全省位置前移，全市经济发展走在全省乃至中部地区前列。

　　－－鄂东特大城市基本建成。全面对接武汉，特大城市格局基本拉开，城市空间布局更加优化，大交通大港口大物流建设取得较大进展，中心城区与大冶城区连为一体，在磁湖、大冶湖之间基本建成一座现代化新城，“四个一”城镇体系[4]基本形成，辐射鄂东皖西赣北的功能充分体现。成功创建国家卫生城市和全国双拥模范城，积极争创中国优秀旅游城市目的地、全国环保模范城市、全国文明城市。

　　－－人民生活水平全面提高。城乡居民生活更加殷实，实现“三高一同步”[5]；社会就业更加充分，住房保障体系全面建立，社会保障全民覆盖；社会事业协调发展，基本公共服务均等化总体实现，城乡居民的思想道德、科学文化、健康素质不断提高；人权得到切实尊重和保障，社会管理体系更加健全，人民群众对自身民主权利、对公共安全的满意度明显提高。

　　－－美丽黄石建设取得重大进展。两型社会建设加快推进，资源循环利用体系基本形成；常用耕地面积保持稳定；单位生产总值能耗和二氧化碳排放大幅下降，主要污染物排放总量显著减少，城乡垃圾无害化处理率达到85%；城乡生态环境质量持续改善，全市森林覆盖率达到37%，城市建成区绿地率达到38%，积极争创国家生态园林城市，夺取中国人居环境奖。

　　实现提前全面建成小康社会的宏伟目标，我们必须统揽全局，把握好以下几条基本原则：第一，坚持以推进资源型城市转型为主线，以经济和产业转型为核心，加快转变经济发展方式，把发展现代服务业放在更加突出的位置，着力提高经济发展的全面性、协调性和可持续性；第二，坚持以招商引资和项目建设为总抓手，着力实施开放先导战略，在扩大开放中加快实施一批大项目、高新技术项目和产业链延伸项目，引进培育更多的市场主体；第三，坚持以改革创新为动力，以更大的决心和勇气推进重点领域和关键环节的改革，加快构建有利于科学发展的体制机制；第四，坚持以城乡统筹为重点，认真落实工业反哺农业、城市支持农村和多予少取放活的方针，大力推进新型城镇化、城乡一体化和基本公共服务均等化，尽快改变农业和农村经济在我市相对落后的局面，实现城乡协调发展；第五，坚持把生态文明建设贯穿全面建设小康社会全过程，深入推进两型社会建设，促进经济社会发展与人口资源环境相协调，努力建设美丽黄石；第六，坚持以保障和改善民生为出发点和落脚点，统筹推进各项社会事业协调发展，努力让人民群众过上更好生活。

　　实现提前全面建成小康社会的宏伟目标，我们将制定落实小康建设三年行动计划总体方案和系列专项方案，重点组织实施好六大工程：一是产业链延伸工程。按照传统型产业补链、成长型产业强链、新兴产业建链的思路，强力推进产业链延伸和集群发展，力争经过3年左右的努力，10大主导产业集群销售收入实现“五个二”目标，即把黑色金属、有色金属打造成2个过千亿元产业，把建材、装备制造打造成2个过500亿元产业，把食品饮料、纺织服装打造成2个过200亿元产业，把精细化工、商贸物流打造成2个过100亿元产业，把生物医药、电子信息打造成2个过50亿元产业；把有色公司培育成千亿元企业，建成6家百亿元企业，每年新增规模以上工业企业80家以上，每年新增上市企业1－2家。大力发展现代服务业，逐年提高服务业在产业结构中的比重。二是城市功能提升工程。坚持新区开发与旧城改造相结合，完成黄石棋盘洲长江大桥、棋盘洲码头、城际铁路、城市铁路网线综合改造和沿江大道、月亮山隧道建设，城区大规模旧城改造、城中村、棚户区改造基本完成，加快推进城镇化建设，城市建成区面积达到100平方公里，城镇化率达到65%左右。三是园区发展工程。全市开发区、园区建成总面积达到150平方公里，完成园区基础设施投入500亿元以上，新增入园工业和服务业企业达到1000家以上，园区生产总值突破1500亿元；力争黄石经济技术开发区成功申报为国家级高新区，大冶经济开发区升格为国家级经济开发区。四是生态文明建设工程。完成磁湖水环境综合治理任务，加强大冶湖治理和保护，确保水面不缩减、水质持续改善；完成黄荆山北麓开山塘口治理任务，南麓开山塘口逐步关闭，新增造林面积15万亩、绿化面积200公顷。五是文化强市工程。深度挖掘矿冶文化，规划、保护和利用好文化遗存，力争黄石矿冶工业遗产成功申报为世界文化遗产，进入国家历史文化名城行列，国家公共文化服务体系示范区全面建成。六是健康黄石工程。建成市中心医院黄金山分院，完成体育场改造，建成市农产品质量安全检测中心和食品药品检测中心。通过三年行动计划尤其是六大工程的实施，为我市提前全面建成小康社会提供有力支撑。

　　三、扎实做好2013年政府工作，为实施三年行动计划、提前全面建成小康社会起好步

　　2013年是实施“十二五”规划承前启后的关键一年，是我市实施“三年行动计划”、实现提前全面建成小康社会的第一年，做好今年的工作，意义重大。今年政府工作的总体要求是：认真贯彻落实党的十八大和中央、全省经济工作会议精神，坚持以邓小平理论、“三个代表”重要思想、科学发展观为指导，按照对接武汉，比学九江，建设鄂东特大城市的思路，深入实施“三大战略”，以推进资源型城市转型跨越为主线，以提高经济增长质量和效益为中心，进一步深化改革开放，进一步强化创新驱动，更加注重经济结构调整，更加注重招商引资和项目建设，更加注重提高城镇化质量，更加注重生态文明建设，更加注重改善民生和创新社会管理，竞进提质，开拓进取，努力实现经济社会平稳较快发展。

　　综合考虑各种因素，2013年经济社会发展的主要预期目标是：地区生产总值增长10.5%以上；规模以上工业增加值增长16%以上；全社会固定资产投资增长25%以上；社会消费品零售总额增长15.5%以上；外商直接投资、外贸出口分别增长15%以上；财政总收入和地方公共财政预算收入分别增长15%以上；居民人均可支配收入增长11%以上；居民消费价格指数控制在4%左右；城镇新增就业4.5万人；城镇登记失业率控制在4.5%以内；人口自然增长率控制在8‰以内。完成省下达的节能降耗和污染减排目标。

　　根据上述总体要求和奋斗目标，今年重点做好以下八个方面的工作：

　　（一）强力推进产业链招商和项目建设

　　突出抓好产业链招商。围绕推进十大主导产业集群发展，编制产业链延伸规划，精心谋划一批补链强链建链项目，明确产业链招商重点，力争引进市外合同资金600亿元以上。建立高位协调的组织领导机构，加强产业链招商队伍建设，提高招商引资组织化水平。建立洽谈、预审、定点、签约、审批项目引进落地机制，确保项目引得进落地快。建立产业链招商和项目建设“六个一”工作推进机制[6]，提高招商引资实效。

　　加大项目建设力度。按照全过程协调服务的要求，建立项目建设领导包保、专题推进、工作督办等机制，着力解决好项目建设中的难题。重点抓好总投资1420亿元、311个亿元以上重大项目的实施，确保185个续建项目完成投资300亿元以上，确保126个计划项目开工建设、完成投资230亿元以上。积极争取国家级承接产业转移示范园区、全国老工业基地调整改造和老工业城区搬迁改造等重大政策，争取中央和省投资份额不低于上年水平。深入推进央企对接工作，力争完成央企签约合作项目5个以上。

　　加快园区建设步伐。坚持产业先导、设施并进、错位发展、机制创新的思路，整体推进园区建设。突出规划引领，高标准编制园区产业发展和基础设施建设规划，在每个省级以上开发区（园区）规划建设一个特色产业园或中小企业园区。推进园区基础设施建设，加快实施黄金山新区东扩南延路网、新港物流（工业）园骨架等重点工程，力争市经济技术开发区百花片5平方公里配套设施全覆盖，大冶经济开发区、阳新经济开发区和西塞山工业园分别完成七通一平面积5平方公里。加快沿江开发建设步伐，推动园区与长江经济带联动发展。加快推进黄石工矿废弃地综合开发试验区规划建设。支持乡镇工业园、工业集中区提档升级，努力壮大镇域经济实力。

　　优化经济发展环境。集中开展优化经济发展环境专项整治行动，严肃查处影响投资发展环境的行为，切实营造重商、安商、护商、亲商的浓厚氛围，让产业第一、企业家老大理念深入人心。制定产业链招商和项目建设新的考核办法，重奖有功单位和人员。推行审批与收费分离改革，对“三类”项目[7]建设费用实行一个口子、一次收清制度。加大用地指标争取力度，加强已供未建用地清理，确保项目建设用地供给。成立项目联合审批办公室，对引进重点项目实行全程代办、一站式服务。加大在招商引资重点区域宣传推介力度，不断提高黄石的知名度和吸引力。

　　（二）深入实施工业强市战略

　　加快产业集群发展。坚持扩大总量与优化结构并举、工业化与信息化深度融合，编制和实施“两计划两工程”[8]行动方案，加快推进沪士电子、光谷联合产业城、重冶模具钢、力丰纺织等一批重点项目建设，进一步壮大支柱产业总量和规模。引导支持关联企业、上下游企业联合重组、攀大靠强，切实盘活企业存量。

　　大力推进创新驱动发展。开展创新型城市创建活动，加快发展装备制造、电子信息等高新技术产业，力争新增高新技术企业10家以上。实施新冶钢特钢产业升级、新兴管业150万吨球墨铸管等一批技改项目，提升传统产业核心竞争力。深化与武汉东湖高新区的对接合作，推进中国光谷·黄石产业园建设。加强企业技术创新能力建设，支持企业申报国家级工程技术研发中心和省级重点实验室，扶持1－2家企业技术中心进入省级以上企业工程技术研发中心行列。深入实施精品名牌战略，加快国家特殊钢产品质量监督检验中心建设，努力争创全国质量强市示范城市。落实人才发展投入政策，加大高层次创新创业人才培养引进力度，促进人才与经济发展深度融合。

　　强化经济运行协调服务。制定并落实新型工业化考核办法，设立工业发展专项资金，扶持主导产业、重点企业及中小微企业等实体经济加快发展。加强经济运行分析调度和预测预警，建立完善重点企业直通车服务、生产要素组织保障联席会议和企业解困挂号销号制度，努力为企业排忧解难。大力培植新的出口主体，加强口岸大通关建设，不断扩大出口规模。

　　（三）加快农业现代化进程

　　推进农业产业化经营。制定和实施农业发展倍增计划。支持劲牌、中粮、雨润等一批国家级省级龙头企业壮大规模，加快形成一批产值过20亿元农产品加工园区、100亿元畜禽水产养殖加工产业集群。推进优质水稻、优质蔬菜、油茶等特色基地建设，促进特色农业规模化发展。加大农业招商引资力度，鼓励发展观光农业、休闲农业。加强和规范土地流转，大力发展农民专业合作社，提高农民市场化组织程度。稳定粮食生产，进一步抓好菜篮子工程建设。

　　改善农村生产生活条件。扎实开展万名干部进万村洁万家活动，强化村庄环境整治，构建垃圾收运处理系统，建设洁化、绿化、美化乡村。加强农田水利基础设施建设，除险加固69座重点小（Ⅱ）型病险水库，综合整治大冶湖主港、阳新木港河等重点河段，抓好12处大型灌溉排水泵站更新改造和塘堰整治，提高灌区排灌和减灾抗旱能力。整镇整村推进基本农田整理，完成农村集体土地所有权登记发证工作。

　　抓好新农村建设和扶贫开发工作。扎实推进新农村建设4个试点镇、36个示范村建设，加快发展农村集体经济，积极推动社会力量参与新农村建设。支持大冶继续抓好城乡一体化省级试点工作。抢抓省里支持幕阜山片区发展与扶贫攻坚的机遇，以完善基础设施、加快产业发展为重点，推进我市新一轮扶贫开发。

　　（四）突破性发展现代服务业

　　推进商贸服务业转型升级。结合旧城改造和新区开发，加快发展购物娱乐、住宿餐饮等传统商贸服务业，推进黄石港和团城山中心商贸区建设。围绕打造社区“8分钟生活圈”，完成中心城区所有农贸市场达标改造。积极引进国内外知名商业品牌，加快建设城市综合体，大力发展大型综合超市、仓储式超市、连锁经营店等新型商贸业态，增强辐射带动能力。实施“限上商贸企业[9]成长工程”，鼓励和支持骨干商贸企业做大做强。健全农村流通网络，繁荣城乡消费市场。

　　积极发展生产性服务业。按照与大工业发展相配套的思路，积极推动制造业企业分离发展现代服务业。加快发展现代物流业，推进新港（棋盘洲）、黄石港（花湖）物流园建设。大力发展第三方物流，培育壮大一批物流龙头企业。积极发展金融保险、信息和中介服务等其它生产性服务业。

　　发展壮大文化旅游产业。发展现代传媒、演艺等文化产业，加强东楚传媒产业园、大冶古建和石雕等产业集聚区建设，提升文化产业核心竞争力。加大矿冶文化推介力度，积极申报国家历史文化名城。加强对重点旅游景区的保护和开发，大力发展红色旅游，支持仙岛湖、东方山创建4A级风景区，支持保安湖国家级湿地公园、黄石港大众山省级森林公园建设，支持西塞山道仕袱古镇重建和西塞神舟会保护，支持铁山打造世界铁城。适应旅游业发展趋势，加快发展自驾游、农家乐等旅游新业态。加强武汉城市圈区域协作和旅游促销，拓展旅游市场，逐步把旅游业培育成带动全市服务业发展的支柱产业。

　　加大服务业发展扶持力度。编制完成全市服务业布局规划，制订出台促进服务业加快发展的政策措施，建立服务业发展考核评价体系。坚持非禁即入、降低门槛、放开搞活的原则，吸引民间资本投资公用事业、文化、教育等行业，促进服务业市场主体的培育。整顿和规范市场秩序，切实维护企业的合法权益，为服务业突破性发展创造宽松的环境。

　　（五）加快建设美丽黄石

　　强化城市规划引领。推进新一轮城市总体规划修编工作。积极开展城市规划“一张图”平台建设[10]，促进规划编制与管理一体化。推进数字黄石基础平台应用及基于3D技术的数字审批系统建设。加强黄石山水空间特色和城市绿道绿楔系统研究，加快编制实施城市公共空间、城市色彩等专项规划，努力提升黄石的生态宜居水平。强化规划在城市建设中的管控作用，切实维护规划的严肃性和权威性。

　　构建综合交通运输体系。建成黄咸高速大冶段、武黄城际铁路黄石段、黄石物流中心，加快推进106国道、棋盘洲港区二期等一批续建工程建设，开工建设棋盘洲大桥连接线、铁山至武汉光谷道路、316国道、黄石新港铁路支线等一批重点工程，力争开工建设棋盘洲长江公路大桥、武穴长江公路大桥及武九客运专线黄石段等一批重点项目，启动首条BRT城市公交专用线和鄂州至咸宁、武汉至南昌高速黄石段及黄石至阳新快速公路等一批项目前期工作，加快形成大路网、大港口、大物流的交通运输体系。

　　加强城乡建设和管理。完善城市功能，启动建设月亮山隧道，推进栗山大道、金山大道东延等道路建设，完成发展大道、苏州路等道路刷黑和河西大道二期改造工程，改造城市道路及人行道2万平方米；加大旧城改造力度，推进一轴、武商摩尔城等中心城区地块改造；加强城市排洪港建设，疏通排水管网60公里，新建天然气管网30公里，开工建设河口污水处理厂，基本完成西塞水厂一期工程，新建和改造供水管网25公里。加快新型城镇化步伐，推进河口韦源口、铁山还地桥两个卫星城基础设施建设，支持还地桥、灵乡、富池开展扩权强镇试点。深入推进国家卫生城市创建工作，确保通过国家检查组的暗访和技术评估。认真做好全省城管大检查迎检工作，力争城管工作在全省位次前移；完善大城管体制，健全数字城管运行、城市管理市场化运作等机制，推进城管进社区、进基层，整体提升城市智能化、精细化管理水平。

　　加快生态文明建设。加强自然生态系统保护，落实最严格的耕地和水资源、森林资源保护制度，加大磁湖、大冶湖等重点水域保护力度，加强应急备用水源建设。抓好城市沿山滨水景观建设和园林绿化工作，新增造林面积5万亩、绿地面积60公顷。加强生态修复治理，推进工矿废弃地复垦利用试点工作，实施重金属污染防治、大气联防联控、矿山地质环境治理等一批重点项目。推进节能减排，扎实开展重点耗能企业能效水平对标活动，继续抓好火电、水泥企业脱硝和钢铁企业脱硫工程建设。大力发展循环经济、低碳经济，支持有色公司申报国家城市矿产示范基地，支持下陆长乐山工业园申报省级循环经济示范园区。

　　（六）切实抓好财税、金融、物价工作

　　提高财税保障和管理水平。认真落实国家、省各项财税政策，推进营改增试点范围扩面，支持企业做大做强，培育壮大后续财源。充分挖掘财政增收潜力，强化对重点税种、重点行业、重点企业税源监控，确保财政收入稳定增长。深化财政预算管理改革，完善财政支出使用绩效评价体系，提高财政资金使用效益和透明度。进一步完善国资监管体系，加强国有资本效益管理，充分发挥国资公司投融资功能，确保国有资本保值增值。

　　加强金融服务体系建设。引导民间资本加大对实体经济、基础设施等领域的投入。积极引进股份制银行、证券、保险、信托等金融机构来我市设立分支机构。支持村镇银行建设，推进农村金融服务全覆盖。加强中小企业担保融资平台建设。创新质押融资、联保贷款等金融产品，不断拓宽企业融资渠道。支持企业开展银行间市场直接融资，推进企业上市和企业债券发行，确保1家企业成功上市。

　　努力保持物价总体水平基本稳定。加强生活必需品储备和市场供应工作，确保重要商品不脱销、不断档。强化物价监管，严厉打击恶意囤积、串通涨价等违法行为。完善社会救助和保障标准与物价上涨联动机制，切实保障低收入群体生活不因价格上涨而降低。

　　（七）着力改善民生和创新社会管理

　　推动更高质量的创业和就业。完善和落实创业培训、小额贷款、税费减免等政策措施，推进创业园（街）、创业孵化基地等创业服务平台建设，实施高层次人才创业孵化、高校毕业生创业引领、返乡农民工回归创业帮扶等三大创业扶持计划，深入推进全民创业。继续做好援企稳岗工作。健全就业援助机制，加强就业指导、信息服务和技能培训，促进大学生、农民工和城镇就业困难人员等重点群体就业。

　　进一步完善城乡社会保障体系。继续深化医药卫生体制改革，全面推进居民医保门诊统筹，确保居民医保政策内住院报销率达到75%。稳步推进我市在武汉大型定点医疗机构医保异地就医联网结算试点，加快推进大冶市、阳新县“金保工程”[11]建设，全面实现“三项保险”[12]市级统筹。强化社会保险扩面征缴，确保退休人员养老金和老工伤人员待遇正常支付。完善劳动关系三方协商机制，加大农民工工资清欠、人力资源市场整顿等监察执法力度，努力构建和谐稳定的劳动关系。大力发展慈善和社会福利事业，启动建设市新福利院。积极探索智能化居家养老模式，推进养老服务社会化、市场化、实体化运营试点。落实殡葬惠民政策，加大非法公墓经营和丧事扰民整治力度，努力倡导社会文明新风。

　　协调发展各项社会事业。坚持教育优先发展，努力办人民满意教育。全面完成学前教育三年行动计划，力争各县（市）区通过省义务教育均衡发展评估验收，加快发展职业技术教育，力促湖北师范学院成功更名湖北师范大学，启动建设青少年校外活动基地。成功建成国家公共文化服务体系示范区。深入推进县级综合公立医院改革，加强基层医疗机构标准化建设，确保所有建制乡镇卫生院和村卫生室建设达标。提高传染病防治和紧急医疗救援水平，积极发展妇幼保健、中医药和康复事业，开工建设市中心医院黄金山分院和食品药品检验检测中心。大力开展全民健身活动，举办黄石市第九届运动会，完成国乒基地重建工程，启动体育场改造，加快建设南湖游泳馆等体育设施。以“智慧城市”[13]建设为目标，推进“三网融合”[14]和十万数字家庭等试点工作。加强人口与计划生育工作，加大出生人口性别比整治力度，提高出生人口素质。

　　加强和创新社会管理。健全党委领导、政府负责、社会协同、公众参与、法治保障的社会管理格局。积极推进“两委、三站、一平台”[15]社区服务体系建设，努力做到信息全收集、服务全到位、管理全覆盖、事态全掌控。建立重点人群特别是失学流浪青少年、农村留守妇女儿童及流动人口、农民工的服务管理机制，提高应对公共危机和突发事件的能力。不断健全群众利益协调、诉求表达、权益保障机制，完善市、县、乡、村四级大调解组织体系，妥善解决群众合法合理诉求。深入推进法治黄石、平安黄石建设，加强社会治安综合治理，健全安全生产和产品质量责任体系，完善食品药品安全监管机制，进一步增强人民群众安全感。扎实推进全国文明城市创建工作，不断提高市民文明素质。

　　（八）切实提高政府执行力

　　实现提前全面建成小康社会的宏伟目标，我们必须勇于担当、敢于负责，以奋发有为的精神状态、务实高效的工作作风，推动各项工作的落实。大力开展政府系统“晋档升级、大步赶超”主题实践活动，对照国内一流标准，比学九江，找差距、定措施，提振精神，全力赶超，使政府系统各部门各单位、各行各业实现争先进位。坚决落实中央改进作风八项规定，切实改进文风会风，大力弘扬真抓实干、求真务实的作风，集中精力抓要事、攻难事、成大事；推行一线工作法，深入基层体察民情，深入一线解决问题。进一步深化行政审批制度改革，精简审批事项，优化审批流程，提高审批效率；加强公务员能力席位建设，强化目标考核管理，建立健全专题推进、激励约束等工作机制，加大行政问责力度，集中整治慵懒散软之风，确保政令畅通。坚持依法行政，严格遵守党风廉政建设各项规定，自觉接受市人大及其常委会的法律监督、工作监督和市政协的民主监督，切实做好人大代表建议案和政协提案办理工作，充分听取各民主党派、工商联、人民团体和社会各界意见。

　　与此同时，认真做好十二五规划实施中期评估和第三次全国经济普查工作。积极支持驻黄部队建设，积极争创全国双拥模范城。继续做好审计、统计、编制、妇女儿童、史志、气象、防震减灾、外事、侨务、对台、保密、档案、民族、宗教、科普和对外援助等工作。

　　今年，我们将重点办好人民群众关心的十件实事：（1）新增城镇就业4.5万人，完成就业创业培训1.5万人；帮扶成功创业6000人，带动就业1.8万人。（2）开工建设和筹集各类保障房5800套，启动棚户区改造3200户；对10000户城镇低收入住房困难家庭发放住房租赁补贴。（3）更新城市公交车100台；完成背街小巷道路改造40条、4万平方米；安装改造背街小巷路灯400盏；实施青山湖2、3、4号湖清淤及处置工程。（4）建成黄石市居家养老呼叫服务中心和10个社区日间照料服务中心。（5）对1000名重度贫困残疾人实施生活补助；为1000名残疾人提供托养服务；免费发放1000件残疾人辅助器具。（6）巩固三万活动成果，实施农村村容村貌连片整治工程；深入推进扶贫开发，减少贫困人口1.8万人，完成200户农村贫困户搬迁。（7）解决10万农村居民及2万农村学校师生饮水不安全问题；免费为农村妇女提供孕前优生健康检查。（8）免费为困难群体和农民工办理法律援助案件1200件、法律援助事项12000件；实施12315消费维权服务站五进（进商场、进超市、进市场、进企业、进景区）项目。（9）创建10所示范幼儿园、50所义务教育标准化学校，更新义务教育学校课桌凳5万套；按高中在校生20%的比例给予家庭经济困难学生国家助学金补助；免费为1000名企业职工提供非全日制中职学历教育。（10）筹集500万元资金，建立大病救助基金，为贫困家庭提供医疗救助。

　　各位代表！新的目标催人奋进，新的征程任重道远。让我们紧密团结在以习近平同志为总书记的党中央周围，在市委的正确领导下，深入贯彻落实科学发展观，加快推进城市转型跨越，为提前全面建成小康社会而努力奋斗！
